# Supplementary material for: High-frequency synthetic apomixis in hybrid rice
Source: Nat Commun. 2022 Dec 27;13:7963. doi: 10.1038/s41467-022-35679-3 (PMC9794695; doi:10.1038/s41467-022-35679-3)
Supplement: Supplementary file 1 — Supplementary Information [file 41467_2022_35679_MOESM1_ESM.pdf]

High-frequency synthetic apomixis in hybrid rice

Vernet *et al.*

Supplementary Table 1. Transformation efficiency in BRS-CIRAD 302.

T-DNA constructs	Co-cultivated calli	Number of HygR cell lines / co-cultivated callus	Resistant cell lines	Regeneration frequency	T0 plants
**T313 : sgMiMe**	22	3.9 +/- 3.8	85	57.9	41
**T314 : sg MiMe _pAtECS:BBM1**	16	2.2 +/- 1	37	70.3	
	32	4.9 +/- 3.9	157	73.8	49
**T315 : sgMiMe _pOsECS:BBM1**	25	3.7 +/- 3.7	95	61.0	
	38	7.9 +/- 7.3	286	56.2	88

Mature seed embryo-derived embryogenic calluses were co-cultivated with *Agrobacterium* EHA105 suspensions carrying either the sgMiMe (T313), the sgMiMe_pAtECS:BBM1 (T314) or the sgMiMe_pOsECS:BBM1 (T315) binary plasmid. Several hygromycin-resistant cell lines were formed per co-cultivated callus. The total number of hygromycin-resistant cell lines is shown: Only a sub-fraction of these lines was tested for regeneration. The number of T0 plants refers to the number of confirmed primary transformants transferred to the greenhouse.

Supplementary Table 2. *OsOSD1* editing efficiencies in primary (T0) transformants of BRS-CIRAD 302.

T-DNA constructs	Biallelic	Homozy-gous	Mono-allelic	WT	Total plants	Edited in *OsOSD1*	Editing efficiency (%)	Number of fertile T0 plants	Fertile plants (%)
**T313 : sgMiMe**	21	14	1	5	41	35	85.4	20	49
**T314 : sg MiMe _pAtECS:BBM1**	27	13	0	9	49	40	81.6	33	67
**T315 : sgMiMe _pOsECS:BBM1**	42	22	5	19	88	64	72.7	36	41
**Total**					178	139	78.1	89	50

T0 plants harbored either the sgMiMe (T313), the sgMiMe_pAtOCS:BBM1 (T314) or the sgMiMe_pOsECS:BBM1 (T315) T-DNA. The lesions observed can be homozygous (same alteration at the two alleles, biallelic (two distinct alterations at the two alleles) or heterozygous (or monoallelic: one allele altered, the other being wild-type (WT)). The number and frequency of fertile plants for each T-DNA construct is shown. The distribution of number of T1 seeds per fertile event is shown in Supplementary Figure 1.

Supplementary Table 3. Lesions observed at CRISPR target regions (red) in *OsOSD1*, *PAIR1* and *OsREC8* coding sequences in fertile BRS-CIRAD 302 primary (T0) transformation events.

T0 Events		Number of T-DNA copies	*Os OSD1* GTGAGAAATTCCGGCGGTAGGGCGGCGCTCGCCGACCCCTCGGGTGGTGGGTTCTTT	*PAIR1* CGCAGTCGCAGTTCTCGCAGGTCTCCCTCGACGACAACCTCCTCACCCTCCTCCCTTCCCCC	*Os REC8* CGTGGGAGTGTGAGTAGGTGGTGTGGCGATCGTGTACGAGAGGAAGGTGAAGGCTCTGTA	T1 seeds
T313: sgMiMe	**T313 4.3**	> 2	Homozygous (+T)	Biallelic (+T/+A)	Bilallelic (+1 / -6)	127
	**T313 9.1**	1	Biallelic (+T/+A)	Biallelic (-2/+1)	Homozygous (-C)	164
	**T313 12.2**	> 2	Biallelic (+1 / -5)	Homozygous (+A)	Biallelic (-3 +1)	227
	**T313 21**	2	Biallelic (-47+46 / +1)	Biallelic (+T/+A)	Biallelic (+1 / -5+1)	168
	**T313 22.1**	1	WT	WT	WT	150
	**T313 22.2**	1	WT	WT	WT	200
T314: sgMiMe _pAtECS:BBM1	**T314 3.1**	1	WT	WT	WT	59
	**T314 12.2**	2	Biallelic (-1/+1)	Biallelic (+T/+A)	Homozygous (+G)	68
	**T314 12.3**	1	WT	WT	WT	664
	**T314 15.1**	> 2	Biallelic (+T/+AG)	Homozygous (+A)	Homozygous (-5+1)	182
	**T314 15.3**	> 2	He (WT/+2)	Homozygous (+A)	Homozygous (--4)	136
	**T314 16**	2	Biallelic (+1 / -8+3)	Homozygous (+A)	Biallelic (-3+G / -2+G)	91
	**T314 23.1**	1	Biallelic (+2 / +1)	Biallelic (+G/+A)	Biallelic (+1 / -24)	31
	**T314 37.7**	2	Biallelic (+1/-5)	Homozygous (+T)	Biallelic (-3/+G)	82
	**T314 37.9**	1	Homozygous (-7)	Biallelic (-2/+1)	Biallelic (-6 / -17)	14
	**T314 44.1**	2	Biallelic (-7 / +1)	Homozygous (+A)	Biallelic (+1 / -2+1)	15
	**T314 44.2**	2	Biallelic (-7 / +1)	Homozygous (+A)	Biallelic (+1 / -6+1)	33
	**T314 46.2**	1	Biallelic (-7 / +1)	Homozygous (+A)	Biallelic (+1 / -5+1)	52
T315: sgMiMe _pOsECS:BBM1	**T315 1.1**	> 2	Homozygous (+2)	Biallelic(-2/+1)	Biallelic (+1 / -5+1)	14
	**T315 3.2**	1	Biallelic (-22 / -34+4)	Homozygous (-28)	Biallelic (-3/-4)	141
	**T315 3.3**	1	Biallelic (-4/-22)	Homozygous (+T)	Bilallelic (-26/+1)	182
	**T315 5.1**	> 2	Homozygous (indel : TA/ AG -19)	Biallelic (+1 / -3)	Homozygous (-5+1)	14
	**T315 5.4**	1	Biallelic (+T/+A)	Biallelic (+G/+T)	Biallelic (+1 / -5)	114
	**T315 5.5**	1	Biallelic (+T/-22)	Biallelic (+G/+T)	Biallelic (+1 / -6+1)	64
	**T315 6.1**	2	Biallelic (+1 / -1)	Heterozygous (WT / -12)	Heterozygous (WT / -35)	86
	**T315 7.2**	2	Biallelic (-19/+14/ del)	Biallelic (+C/+A)	Biallelic (-24 / +1)	150
	**T315 7.4**	> 2	Biallelic (-19+14 / -21+90)	Biallelic (+C/+A)	Biallelic (-4 / -24)	73
	**T315 8.1**	1	Homozygous (-5)	Homozygous (+G)	Homozygous (+C)	264
	**T315 8.2**	1	Biallelic (-5/+A)	Homozygous (+G)	Homozygous (+C)	195
	**T315 8.3**	1	Biallelic (-5/+A)	Homozygous (+G)	Homozygous (+C)	123
	**T315 14.1**	1	WT	WT	WT	9
	**T315 14.2**	1	WT	WT	WT	23
	**T315 14.3**	1	WT	WT	WT	73
	**T315 16.1**	> 2	Biallelic (+1/-2)	Biallelic (-A/+A)	Biallelic(-15 / -5)	18
	**T315 16.3**	2	Homozygous (+1)	Biallelic (+T/+A)	Biallelic (-2 / -4)	14
	**T315 21.1**	1	WT	WT	WT	55
	**T315 24.1**	> 2	Homozygous (-29)	Biallelic (-2/+1)	Biallelic (-4 / -4+1)	50
	**`T315 31.1**	> 2	Biallelic (-39+2 / -7)	Homozygous (-A)	Biallelic (-6 / -6+3)	24
	**T315 31.4**	1	Biallelic (-2/+1)	Biallelic (+1/-7)	Biallelic (+1 / -24)	36
	**T315 41.2**	1	Biallelic (+T/+A)	Biallelic (+1 / -4/+3)	Biallelic (-5 / -5+1)	30
	**T315 41.6**	> 2	Homozygous (+1)	Biallelic (+T/+A)	Biallelic (-4+1)	25

T0 events harbored the sgMiMe (T313), the sgMiMe_pAtECS:BBM1 (T314) or the sgMiMe_pOsECS:BBM1 (T315) T-DNAs. Homozygous and biallelic lesions are highlighted by different green background colors. Alleles exhibiting inserted or deleted nucleotides that diverge from a multiple of three are indicative of frameshift mutations and are likely inactivated. Wild-type (WT) or heterozygous target regions appear on an orange background. The number of seeds harvested from T0 plants is shown. For T313, only the six T0 events exhibiting the highest number of T1 seeds were analyzed. For T314 and T315 only the T0 events showing more than nine T1 seeds were analyzed. The T314 15.3 line is heterozygous at the OsOSD1 locus and homozygous mutant at PAIR1 and OsREC8. Such a mutation configuration should lead to plant sterility. However, sequencing of T1 progeny plants proved that a late mutation (-31 nt deletion + 3nt insertion) eventually occurred in the primary transformant, altering the remaining intact allele of OsOSD1 after T0 leaf sample collection for DNA analysis. Line T315 6.1 is a putative knock-out in OsOSD1 only, which is known to be fertile[1].

Supplementary Table 4. Detail of distribution of ploidy level among T2 progenies.

T0 event	T-DNA copy	T1 plant	2n	4n	% Diploids	Reminder % diploids in T1s
**T314 15.1**	>2	T314 15.1/4	45	2	96	
		T314 15.1/6	40	1	98	
		T314 15.1/8	40	0	100	
		T314 15.1/10	40	2	95	
		T314 15.1/11	45	2	96	
			210	7	96.8	98
**T314 37.7**	2	T314 37.7/11	42	6	87	
		T314 37.7/14	41	10	80	
		T314 37.7/15	40	2	95	
		T314 37.7/19	37	4	90	
		T314 37.7/20	44	2	96	
			204	24	89.5	92
**T315 3.2**	1	T315 3.2/485	38	6	86	
		T315 3.2/492	40	4	91	
		T315 3.2/501	40	4	91	
		T315 3.2/503	44	0	100	
		T315 3.2/504	43	1	99	
			205	15	93.2	96
**T315 5.4**	1	T315 5.4/409	47	1	98	
		T315 5.4/411	43	1	98	
		T315 5.4/412	41	3	93	
		T315 5.4/414	60	6	91	
		T315 5.4/417	44	2	96	
			235	13	94.8	93
**T315 8.1**	1	T315 8.1/292	40	0	100	
		T315 8.1/313	51	5	91	
		T315 8.1/318	39	1	97	
		T315 8.1/322	38	2	95	
		T315 8.1/329	33	7	82	
			201	15	93.1	95
**T315 8.2**	1	T315 8.2/207	50	10	83	
		T315 8.2/211	39	7	84	
		T315 8.2/212	54	10	84	
		T315 8.2/234	56	3	95	
		T315 8.2/350	40	4	91	
			239	34	87.5	97

Each T1 progeny is formed by five T1 plants of the T314 (sgMiMe_pAtECS:BBM1) 15.1 and 37.7 events, and of the T315 (sgMiMe_pOsECS:BBM1) 3.2, 5.4, 8.1 and 8.2 events. Frequency of diploid plants at the T1 generation is provided for reference.

Supplementary Table 5. Detail of distribution of ploidy level among T3 progenies.

	2n	4n	% diploids
T314 15.1 / 11 / 7	98	2	98
T314 15.1 / 6 / 10	99	1	99
T314 15.1 / 8 / 7	99	1	99
			98.7
T314 37.7 / 19 / 4	93	7	93
T314 37.7 / 20 /2	92	8	92
T314 37.7 / 11/ 6	96	4	96
			93.7

T315 8.1/ 181 /4	94	6	94
T315 8.1/ 182 /10	92	8	92
T315 8.1 /186/3	92	8	92
			92.7
T315 5.4/ 66/10	96	4	96
T315 5.4/ 70/4	98	2	98
T315 5.4/ 67/7	96	4	96
			96.7

One hundred T3 progeny plants of each of three randomly selected T2 plants of T314 (sgMiMe_pAtECS:BBM1) 15.1 and 37.7 events, and T315 (sgMiMe_pOsECS:BBM1) 5.4 and 8.1 events have been analyzed

Supplementary Table 6. Analysis of fertile Kitaake primary (T0) transgenics harboring either the *sgMiMe_ pAtECS:BBM1* (T314) T-DNA or the *sgMiMe_pOsECS:BBM1* (T315) T-DNA.

A.

T0 Events		*OSD1* gRNA1	*OSD1* gRNA2	*PAIR1*	*REC8*
		AAGTGAGAAATTCCGGCGGTAGGGCG	CGGCGCTCGCCGACCCCTCGGGTGGT	CCTCGACGACAACCTCCTCACCCT	TGGTGTGGCGATCGTGTACGAGAG
T314:sgMiMe-pAtECS:BBM1	#3a	WT	Biallelic (-2/+2)	Biallelic (-2/+1)	Biallelic (-13/-14)
	#33	Heterozygous (-3)	Biallelic (-2/+1)	Homozygous (+A)	Biallelic (-2/-2)
T315:sgMiMe-pBOsECS:BBM1	#9a	WT	Biallelic (+2/+1)	Biallelic (-1/+1)	Homozygous (-5)
	27d	WT	Homozygous (+T)	Biallelic (-1/+1, 1bp substitution)	Biallelic (-4/-5)

B.

Line #	# T1 Progeny	Diploids	Tetraploids	Apomictic/ diploid progeny (%)	Panicle fertility (from master tillers of 5 T1 plants)
Wild-type control					89.7 %
3A	69 (6 twins)	38	31	55	81.5 %
33	61 (10 twins)	51	10	84	74.1%

C.

Line #	# T1 Progeny	Diploids	Tetraploids	Apomictic/ diploid progeny %age	Panicle fertility (from master tillers of 5 T1 plants)
9A	55 (1 twin)	3	52	5	82.0 %
27D	58 (6 twins)	49	9	84	59.1%

A. Mutations at the *CRISPR*-Cas9 target sites in *OSD1*, *PAIR1* and *REC8* genes. All the lines analyzed were confirmed to be frame shift loss-of-function mutations for all the alleles of the three *MiMe* genes. Line #33 for *sgMiMe_pAtECS:BBM1* construct was the only line analyzed in which *OSD1* had a 3 bp deletion in one of the alleles at gRNA1 site. The same alleles had 1 bp insertion at the gRNA2 site. Protospacer sequences are in red. **B-C**. Fertility and apomixis efficiency in transgenic Kitaake plants generated using the sgMiMe_pAtECS:BBM1 (T314) (**A**) and sgMiMe_pOsECS:BBM1 (T315) (**B**) T-DNA vectors.

Supplementary Table 7 . Detail of traits scored in T2 progeny plants of five T1 plants.

T0 Event/T1 plant	T2/F1 plant number	Plant height	Number of tillers	n-1 Leaf length	n-1 Leaf width	Flag leaf length	Flag leaf width	Main panicle lentgh
T314 15.1/4	8	84.3 a	12.9 a	47.8 a	1.4 a	27.1 a	1.6 a	24.4 a
T314 15.1/6	8	83.9 a	14.1 a	49.9 a	1.5 a	28,1 a	1.6 a	24.3 a
T314 15.1/8	6	83.8 a	15.5 a	54.3 a	1.1 a	27.7 a	1.4 a	25.4 a
T314 15.1/10	5	81.0 a	13.4 a	42.9 a	1.5 a	31.6 a	1,7 a	24.1 a
T314 15.1/11	9	80.6 a	16.0 a	47.1 a	1.3 a	27.3 a	1.5 a	23.8 a
T314 15.1 mean		82.5	14.5	47.3	1.4	28.3	1.6	24.2
T314 37.7/11	7	85.7 a	15.4 a	45.5 a	1.4 a	26.0 a	1.5 a	23.9 a
T314 37.7/14	8	85.4 a	16.3 a	45.4 a	1.4 a	27.6 a	1.5 a	23.3 a
T314 37.7/15	8	85.0 a	14.1 a	49.2 a	1.4 a	28.9 a	1.5 a	23.8 a
T314 37.7/19	8	82.9 a	15.9 a	45.9 a	1.3 a	26.2 a	1.5 a	23.6 a
T314 37.7/20	9	85.9 a	14.2 a	44.3 a	1.4 a	25.7 a	1.4 a	23.6 a
T314 37.7 mean		85.0	15.2	46.0	1.4	26.9	1.5	23.6
T315 5.4/63	7	85.0 a	15.4 a	45.4 a	1.5 a	25.9 a	1.5 a	24.4 a
T315 5.4/66	5	87.4 a	12.6 a	45.6 a	1.5 a	26.4 a	1.6 a	24.5 a
T315 5.4/67	10	89.1 a	13.8 a	43.9 a	1.5 a	23.8 a	1.6 a	24.6 a
T315 5.4/70	8	87.3 a	14.3 a	48.1 a	1.7 a	27.1 a	1.7 a	24.6 a
T315 5.4/86	8	88.0 a	14.6 a	49.1 a	1.6 a	26.9 a	1.8 a	25.3 a
T315 5.4 mean		87.3	14.4	46.5	1.6	26.0	1.6	24.6
T315 8.1/181	8	83.4 a	14.0 a	48.4 a	1.4 a	27.8 a	1.6 a	24.8 a
T315 8.1/182	8	82.1 a	13.9 a	47.0 a	1.5 a	27.5 a	1.6 a	24.4 a
T315 8.1/186	5	81.5 a	14.2 a	51.1 a	1.6 a	29.6 a	1.7 a	26.1 a
T315 8.1/188	5	86.8 a	13.4 a	48.0 a	1.4 a	28.0 a	1.6 a	26.2 a
T315 8.1/189	8	83.3 a	13.5 a	48.3 a	1.5 a	30.2 a	1.6 a	26.1 a
T315 8.1 mean		83.2	13.9	48.5	1.5	28.6	1.6	25.3
BRS-CIRAD 302 mean	15	84.8	13.4	50.1	1,3	31.9	1.6	24.5

T2 progenies of T314 (sgMiMe_pAtECS:BBM1) 15.1 and 37.7 events and T315 (sgMiMe_pOsECS:BBM1) 5.4 and 8.1 events have been evaluated. Variation across the five T2 progenies of each event was individually examined for statistical significance using a Kruskal-Wallis test. Numbers followed by the same letter are not statistically different at the α-risk of 0.05.

Supplementary Table 8. Pollen viability estimated using Alexander's staining method in BRS-CIRAD 302 F1 hybrid plants and T2 progeny plants of 4 apomictic events.

Material	Plants	Average pollen viability	SD
BRS-CIRAD 302	4	59.1	7.0
T2 T314 15.1	4	85.9	2.3
T2 T314 37.7	6	95.3	1.7
T2 T315 5.4	5	95.2	1.7
T2 T315 8.1	4	94.6	1.9

BRS-CIRAD 302 control plants and T2 plants of T314 (sgMiMe_pAtECS:BBM1) 15.1 and 37.7 events and T315 (sgMiMe_pOsECS:BBM1) 5.4 and 8.1 events were grown under the same controlled greenhouse conditions and flowered synchronously. At least four distinct plants were used and frequency was established by counting at least 1,500 pollen grains released from the anthers of two distinct flowers per plant.

Supplementary Table 9. Seed shape traits of dehulled mature seeds collected on BRS-CIRAD 302 F1 hybrid (F2 seeds), BRS-CIRAD 302 parental lines (1F and D24) and T2 plants of 4 apomictic lines (T3 seeds).

	length (mm)	width (mm)	volume (mm3)	shape
1F	6.88	2.11	10.18	3.26
D24	7.32	2.01	9.79	3.66
BRS-CIRAD 302	7.17	2.19	11.36	3.28
F2 seeds	7.18 a,b	2.11 a	10.58 a,b	3.42 a
T314 15.1	7.15 a,b	2.05 a,b	9.97 a,b	3.50 a
T314 37.7	6.95 b	2.02 b	9.39 b	3.45 a
T315 5.4	7.37 a	2.12 a	10.97 a	3.48 a
T315 8.1	7.33 a	2.08 a,b	10.46 a,b	3.54 a
Average apomictics	7.18	2.06	10.13	3.49

Seed shape traits of BRS-CIRAD 302 commercial F1 seeds are shown for comparison. Numbers followed by the same letter are not significantly different from each other in a Kruskal-Wallis test (α-risk : 0.05). Source data are provided as a Source Data file.

Supplementary Table 10. Target sequences in *OsOSD1*, *PAIR1* and *OsREC8* genes.

sgRNA	5’- 3’ target sequence **(PAM)**
sgRNA OsOSD1/1	GAGAAATTCCGGCGGTAGGG **CGG**
sgRNA OsOSD1/2	GCGCTCGCCGACCCCTCGGG **TGG**
sgRNA PAIR1	GGTGAGGAGGTTGTCGTCGA **GGG**
sgRNA OsREC8	GTGTGGCGATCGTGTACGAG **AGG**

Supplementary Table 11. list of primers used for the molecular characterization of transformants and SSR genotyping.

Primer	5'-3' Primer sequence	Comments
REG1-F<br>REG1-R	ATGGAATGATGGATGAATGTTCAC<br>ATGGAATGATGGATGAATGTTCAC	Amplification of OsECA1 promoter region
OsOSD1-F<br>OsOSD1-R	TTACTTGGAAGAGGCAGGAGCC<br>ACCTTGACGACTGACGTGATGTC	Amplification of sgOsOSD1 target site
PAIR1-F<br>PAIR1-R	GTGGTGTGGTGTGTTCAGGAG<br>TGGAATCCCCAATCAGTAAGGCAC	Amplification of sgPAIR1 target site
OsREC8-F<br>OsREC8-R	GCACTAAGGCTCTCCGGAATTCTC<br>AATGGATCAAGGAGGAGGCACC	Amplification of sgOsREC8 target site
pEC1.2:BBM1-F<br>pEC1.2:BBM1-R	TTCCCTTTCCCACACGCTAC<br>AGAAGGAGGAGGAGGAGAAGC	Ascertaining presence of the pEC1.2:BBM1 cassette
pECA1:BBM1-F<br>pECA1:BBM1-R	ACCGTCAATCCTTTCCCATTCCTC<br>GAAGTCCTCCAGCTTCGGC	Ascertaining presence of the pECA1:BBM1 cassette
Cas9-F<br>Cas9-R	TGCCTGCGGAGGATAGCATGAAGCTC<br>TACCACGAGAAGTACCCGACCATCT	Ascertaining presence of Cas9
HPT-F<br>HPT-R	CTGAACTCACCGCGACGTCTG<br>GGCGTCGGTTTCCACTATCG	Determination of T-DNA copy number integrated in T0 plants
RM1-F<br>RM1-R	GCGAAAACACAATGCAAAAA<br>GCGTTGGTTGGACCTGAC	Amplification of SSR locus RM1 located on chr 1
RM25-F<br>RM25-R	GGAAAGAATGATCTTTTCATGG<br>CTACCATCAAAACCAATGTTC	Amplification of SSR locus RM25 located on chr 8
RM215-F<br>RM215-R	CAAAATGGAGCAGCAAGAGC<br>TGAGCACCTCCTTCTCTGTAG	Amplification of SSR locus RM215 located on chr 9
RM287-F<br>RM287-R	TTCCCTGTTAAGAGAGAAATC<br>GTGTATTTGGTGAAAGCAAC	Amplification of SSR locus RM287 located on chr 11

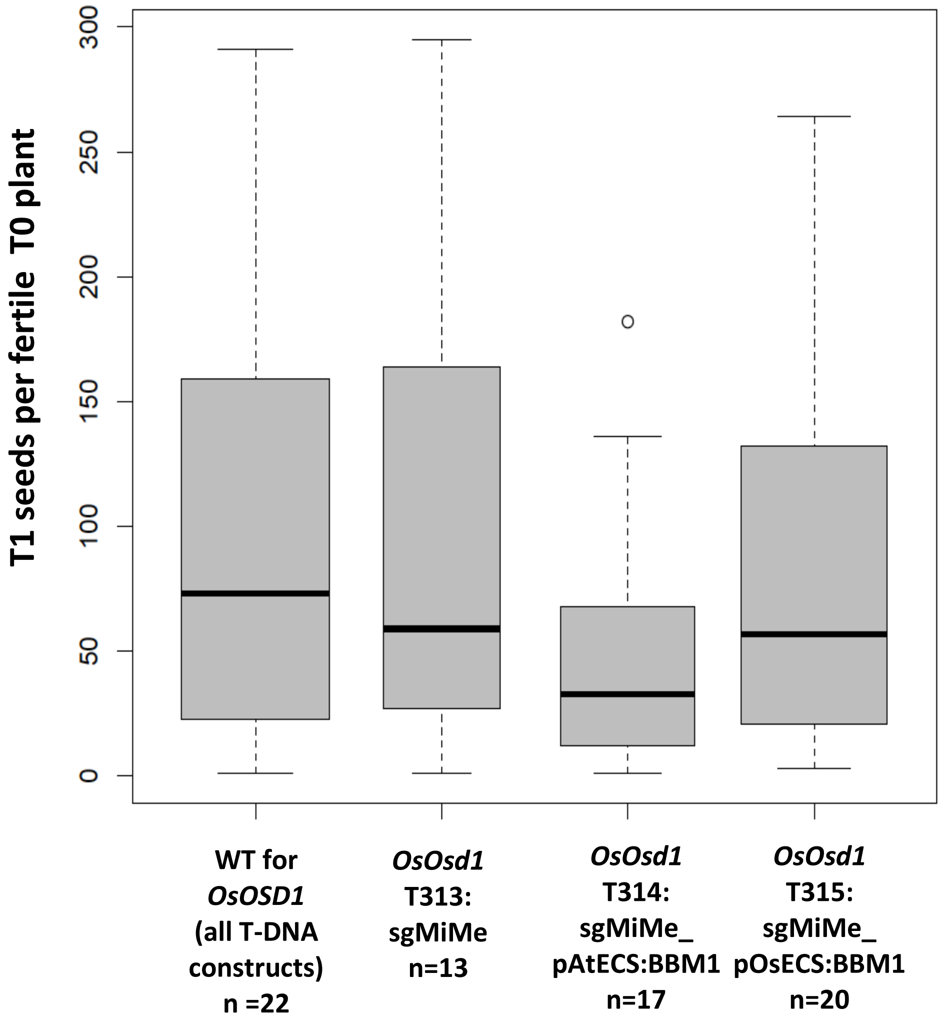

Supplementary Figure 1. Distribution of the number of seeds harvested on fertile T0 plants. From left to right: transformed but unedited for *OsOSD1* (pooled T313, T314, and T315 events); harboring the T313 sgMiMe T-DNA and edited for *OsOSD1*; harboring the T314 sgMiMe-pAtECS:BBM1 T-DNA and edited for *OsOSD1*; and harboring the T314 sgMiMe-pOsECS:BBM1 T-DNA and edited for *OsOSD1*. The line dividing the box is the median whereas the box represents the middle 50% of the group and the upper and lower edges of the box represents third and first quartile limits respectively. Upper and lower whiskers represent scores outside the middle 50%. Outliers position outside whiskers

A

	RM1		RM25		RM215		RM287	
	Allele 1	Allele 2	Allele 1	Allele 2	Allele 1	Allele 2	Allele 1	Allele 2
1F parent	119	119	162	162	167	167	134	134
1F parent	119	119	162	162	167	167	134	134
D24 parent	125	125	164	164	163	163	132	132
D24 parent	125	125	164	164	163	163	132	132
BRS-CIRAD 302	119	125	162	164	163	167	132	134
BRS-CIRAD 302	119	125	162	164	163	167	132	134
BRS-CIRAD 302	119	125	162	164	163	167	132	134
F2 progeny 1	119	125	162	164	163	167	132	134
F2 progeny 2	119	125	162	164	163	167	132	134
F2 progeny 3	119	125	162	164	163	163	132	134
F2 progeny 4	119	119	162	164	163	167	134	134
F2 progeny 5	119	125	162	164	163	167	134	134
F2 progeny 6	119	119	162	164	163	167	134	134
F2 progeny 7	125	125	164	164	163	163	134	134
F2 progeny 8	119	125	164	164	167	167	134	134
F2 progeny 9	119	125	164	164	163	167	134	134
F2 progeny 10	119	125	162	162	167	167	132	132
F2 progeny 11	119	119	162	162	167	167	134	134
F2 progeny 12	119	125	164	164	163	163	132	132

B

T313 12.1 1	119	125	162	164	163	167	132	134
T313 12.1 2	119	125	162	164	163	167	132	134
T313 12.1 3	119	125	162	164	163	167	132	134
T313 12.1 4	119	125	162	164	163	167	132	134
T313 12.1 5	119	125	162	164	163	167	132	134
T313 12.1 6	119	125	162	164	163	167	132	134
T313 12.1 7	119	125	162	164	163	167	132	134
T313 12.1 8	119	125	162	164	163	167	132	134
T313 12.1 9	119	125	162	164	163	167	132	134
T313 12.1 10	119	125	162	164	163	167	132	134
T313 21 1	119	125	162	164	163	167	132	134
T313 21 2	119	125	162	164	163	167	132	134
T313 21 3	119	125	162	164	163	167	132	134
T313 21 4	119	125	162	164	163	167	132	134
T313 21 5	119	125	162	164	163	167	132	134
T313 21 6	119	125	162	164	163	167	132	134
T313 21 7	119	125	162	164	163	167	132	134
T313 21 8	119	125	162	164	163	167	132	134
T313 21 9	119	125	162	164	163	167	132	134
T313 21 10	119	125	162	164	163	167	132	134

C

T314 15.1 1	119	125	162	164	163	167	132	134
T314 15.1 2	119	125	162	164	163	167	132	134
T314 15.1 3	119	125	162	164	163	167	132	134
T314 15.1 4	119	125	162	164	163	167	132	134
T314 15.1 5	119	125	162	164	163	167	132	134
T314 15.1 6	119	125	162	164	163	167	132	134
T314 15.1 7	119	125	162	164	163	167	132	134
T314 15.1 8	119	125	162	164	163	167	132	134
T314 15.1 9	119	125	162	164	163	167	132	134
T314 15.1 10	119	125	162	164	163	167	132	134
T314 15.1 11	119	125	162	164	163	167	132	134
T314 15.1 12	119	125	162	164	163	167	132	134
T314 15.1 13	119	125	162	164	163	167	132	134
T314 15.1 14	119	125	162	164	163	167	132	134
T314 15.1 15	119	125	162	164	163	167	132	134
T314 15.1 16	119	125	162	164	163	167	132	134
T314 15.1 17	119	125	162	164	163	167	132	134
T314 15.1 18	119	125	162	164	163	167	132	134
T314 15.1 19	119	125	162	164	163	167	132	134
T314 15.1 20	119	125	162	164	163	167	132	134
T314 15.1 21	119	125	162	164	163	167	132	134
T314 15.1 22	119	125	162	164	163	167	132	134
T314 15.1 23	119	125	162	164	163	167	132	134
T314 15.1 24	119	125	162	164	163	167	132	134
T314 15.1 25	119	125	162	164	163	167	132	134
T314 15.1 26	119	125	162	164	163	167	132	134
T314 15.1 27	119	125	162	164	163	167	132	134
T314 15.1 28	119	125	162	164	163	167	132	134
T314 15.1 29	119	125	162	164	163	167	132	134
T314 15.1 30	119	125	162	164	163	167	132	134
T314 15.1 31	119	125	162	164	163	167	132	134
T314 15.1 32	119	125	162	164	163	167	132	134

D

T315_5.4_-63_B-_1	119	125	162	164	163	167	132	134
T315_5.4_-63_B-_10	119	125	162	164	163	167	132	134
T315_5.4_-63_B-_11	119	125	162	164	163	167	132	134
T315_5.4_-63_B-_12	119	125	162	164	163	167	132	134
T315_5.4_-63_B-_13	119	125	162	164	163	167	132	134
T315_5.4_-63_B-_14	119	125	162	164	163	167	132	134
T315_5.4_-63_B-_15	119	125	162	164	163	167	132	134
T315_5.4_-63_B-_16	119	125	162	164	163	167	132	134
T315_5.4_-63_B-_17	119	125	162	164	163	167	132	134
T315_5.4_-63_B-_18	119	125	162	164	163	167	132	134
T315_5.4_-63_B-_19	119	125	162	164	163	167	132	134
T315_5.4_-63_B-_2	119	125	162	164	163	167	132	134
T315_5.4_-63_B-_20	119	125	162	164	163	167	132	134
T315_5.4_-63_B-_3	119	125	162	164	163	167	132	134
T315_5.4_-63_B-_4	119	125	162	164	163	167	132	134
T315_5.4_-63_B-_5	119	125	162	164	163	167	132	134
T315_5.4_-63_B-_6	119	125	162	164	163	167	132	134
T315_5.4_-63_B-_7	119	125	162	164	163	167	132	134
T315_5.4_-63_B-_8	119	125	162	164	163	167	132	134
T315_5.4_-63_B-_9	119	125	162	164	163	167	132	134
T315_5.4_-66_B-_1	119	125	162	164	163	167	132	134
T315_5.4_-66_B-_10	119	125	162	164	163	167	132	134
T315_5.4_-66_B-_11	119	125	162	164	163	167	132	134
T315_5.4_-66_B-_12	119	125	162	164	163	167	132	134
T315_5.4_-66_B-_13	119	125	162	164	163	167	132	134
T315_5.4_-66_B-_14	119	125	162	164	163	167	132	134
T315_5.4_-66_B-_15	119	125	162	164	163	167	132	134
T315_5.4_-66_B-_16	119	125	162	164	163	167	132	134
T315_5.4_-66_B-_17	119	125	162	164	163	167	132	134
T315_5.4_-66_B-_18	119	125	162	164	163	167	132	134
T315_5.4_-66_B-_19	119	125	162	164	163	167	132	134
T315_5.4_-66_B-_2	119	125	162	164	163	167	132	134
T315_5.4_-66_B-_20	119	125	162	164	163	167	132	134
T315_5.4_-66_B-_21	119	125	162	164	163	167	132	134
T315_5.4_-66_B-_22	119	125	162	164	163	167	132	134
T315_5.4_-66_B-_3	119	125	162	164	163	167	132	134
T315_5.4_-66_B-_4	119	125	162	164	163	167	132	134
T315_5.4_-66_B-_5	119	125	162	164	163	167	132	134
T315_5.4_-66_B-_6	119	125	162	164	163	167	132	134
T315_5.4_-66_B-_7	119	125	162	164	163	167	132	134
T315_5.4_-66_B-_8	119	125	162	164	163	167	132	134
T315_5.4_-66_B-_9	119	125	162	164	163	167	132	134
T315_5.4_-67_B-_1	119	125	162	164	163	167	132	134
T315_5.4_-67_B-_10	119	125	162	164	163	167	132	134
T315_5.4_-67_B-_11	119	125	162	164	163	167	132	134
T315_5.4_-67_B-_12	119	125	162	164	163	167	132	134
T315_5.4_-67_B-_13	119	125	162	164	163	167	132	134
T315_5.4_-67_B-_14	119	125	162	164	163	167	132	134
T315_5.4_-67_B-_15	119	125	162	164	163	167	132	134
T315_5.4_-67_B-_16	119	125	162	164	163	167	132	134
T315_5.4_-67_B-_17	119	125	162	164	163	167	132	134
T315_5.4_-67_B-_18	119	125	162	164	163	167	132	134
T315_5.4_-67_B-_19	119	125	162	164	163	167	132	134
T315_5.4_-67_B-_2	119	125	162	164	163	167	132	134
T315_5.4_-67_B-_20	119	125	162	164	163	167	132	134
T315_5.4_-67_B-_22	119	125	162	164	163	167	132	134
T315_5.4_-67_B-_23	119	125	162	164	163	167	132	134
T315_5.4_-67_B-_24	119	125	162	164	163	167	132	134
T315_5.4_-67_B-_25	119	125	162	164	163	167	132	134
T315_5.4_-67_B-_3	119	125	162	164	163	167	132	134
T315_5.4_-67_B-_4	119	125	162	164	163	167	132	134
T315_5.4_-67_B-_5	119	125	162	164	163	167	132	134
T315_5.4_-67_B-_7	119	125	162	164	163	167	132	134
T315_5.4_-67_B-_8	119	125	162	164	163	167	132	134
T315_5.4_-67_B-_9	119	125	162	164	163	167	132	134
T315_5.4_-70_B-_1	119	125	162	164	163	167	132	134
T315_5.4_-70_B-_10	119	125	162	164	163	167	132	134
T315_5.4_-70_B-_11	119	125	162	164	163	167	132	134
T315_5.4_-70_B-_12	119	125	162	164	163	167	132	134
T315_5.4_-70_B-_13	119	125	162	164	163	167	132	134
T315_5.4_-70_B-_14	119	125	162	164	163	167	132	134
T315_5.4_-70_B-_15	119	125	162	164	163	167	132	134
T315_5.4_-70_B-_16	119	125	162	164	163	167	132	134
T315_5.4_-70_B-_17	119	125	162	164	163	167	132	134
T315_5.4_-70_B-_18	119	125	162	164	163	167	132	134
T315_5.4_-70_B-_19	119	125	162	164	163	167	132	134
T315_5.4_-70_B-_2	119	125	162	164	163	167	132	134
T315_5.4_-70_B-_20	119	125	162	164	163	167	132	134
T315_5.4_-70_B-_21	119	125	162	164	163	167	132	134
T315_5.4_-70_B-_22	119	125	162	164	163	167	132	134
T315_5.4_-70_B-_3	119	125	162	164	163	167	132	134
T315_5.4_-70_B-_4	119	125	162	164	163	167	132	134
T315_5.4_-70_B-_5	119	125	162	164	163	167	132	134
T315_5.4_-70_B-_6	119	125	162	164	163	167	132	134
T315_5.4_-70_B-_7	119	125	162	164	163	167	132	134
T315_5.4_-70_B-_8	119	125	162	164	163	167	132	134
T315_5.4_-70_B-_9	119	125	162	164	163	167	132	134
T315_5.4_-86_B-_1	119	125	162	164	163	167	132	134
T315_5.4_-86_B-_10	119	125	162	164	163	167	132	134
T315_5.4_-86_B-_11	119	125	162	164	163	167	132	134
T315_5.4_-86_B-_12	119	125	162	164	163	167	132	134
T315_5.4_-86_B-_13	119	125	162	164	163	167	132	134
T315_5.4_-86_B-_14	119	125	162	164	163	167	132	134
T315_5.4_-86_B-_15	119	125	162	164	163	167	132	134
T315_5.4_-86_B-_16	119	125	162	164	163	167	132	134
T315_5.4_-86_B-_17	119	125	162	164	163	167	132	134
T315_5.4_-86_B-_18	119	125	162	164	163	167	132	134
T315_5.4_-86_B-_19	119	125	162	164	163	167	132	134
T315_5.4_-86_B-_2	119	125	162	164	163	167	132	134
T315_5.4_-86_B-_20	119	125	162	164	163	167	132	134
T315_5.4_-86_B-_21	119	125	162	164	163	167	132	134
T315_5.4_-86_B-_22	119	125	162	164	163	167	132	134
T315_5.4_-86_B-_23	119	125	162	164	163	167	132	134
T315_5.4_-86_B-_24	119	125	162	164	163	167	132	134
T315_5.4_-86_B-_25	119	125	162	164	163	167	132	134
T315_5.4_-86_B-_26	119	125	162	164	163	167	132	134
T315_5.4_-86_B-_3	119	125	162	164	163	167	132	134
T315_5.4_-86_B-_4	119	125	162	164	163	167	132	134
T315_5.4_-86_B-_5	119	125	162	164	163	167	132	134
T315_5.4_-86_B-_6	119	125	162	164	163	167	132	134
T315_5.4_-86_B-_7	119	125	162	164	163	167	132	134
T315_5.4_-86_B-_8	119	125	162	164	163	167	132	134
T315_5.4_-86_B-_9	119	125	162	164	163	167	132	134

Supplementary Figure 2. Genotypes of T1 and T2 progenies along with BRS-CIRAD 302 parental lines, BRS-CIRAD 302 F1 hybrid and sexual F2 progenies.

Four microsatellites (SSR) loci (RM1; RM25; RM215; RM287) were used for genotyping. **A**. Homozygous genotype of the 1F and D24 parents and heterozygous genotype of the BRS-CIRAD 302 hybrid. Twelve F2 progenies showing free segregation of parental alleles. **B.** Representative T1 progenies (n=10) of two T0 T313 sgMiMe plants (events 12.1 and 21) exhibiting no marker segregation**. C.** Representative T314 sgMiMe_pAtECS:BBM1 T1 progeny (event 15.1) (n=32) exhibiting no marker segregation. **D.** Representative T315 sgMiMe_pOsECS:BBM1 T2 progeny plants (event 5.4) exhibiting no marker segregation.

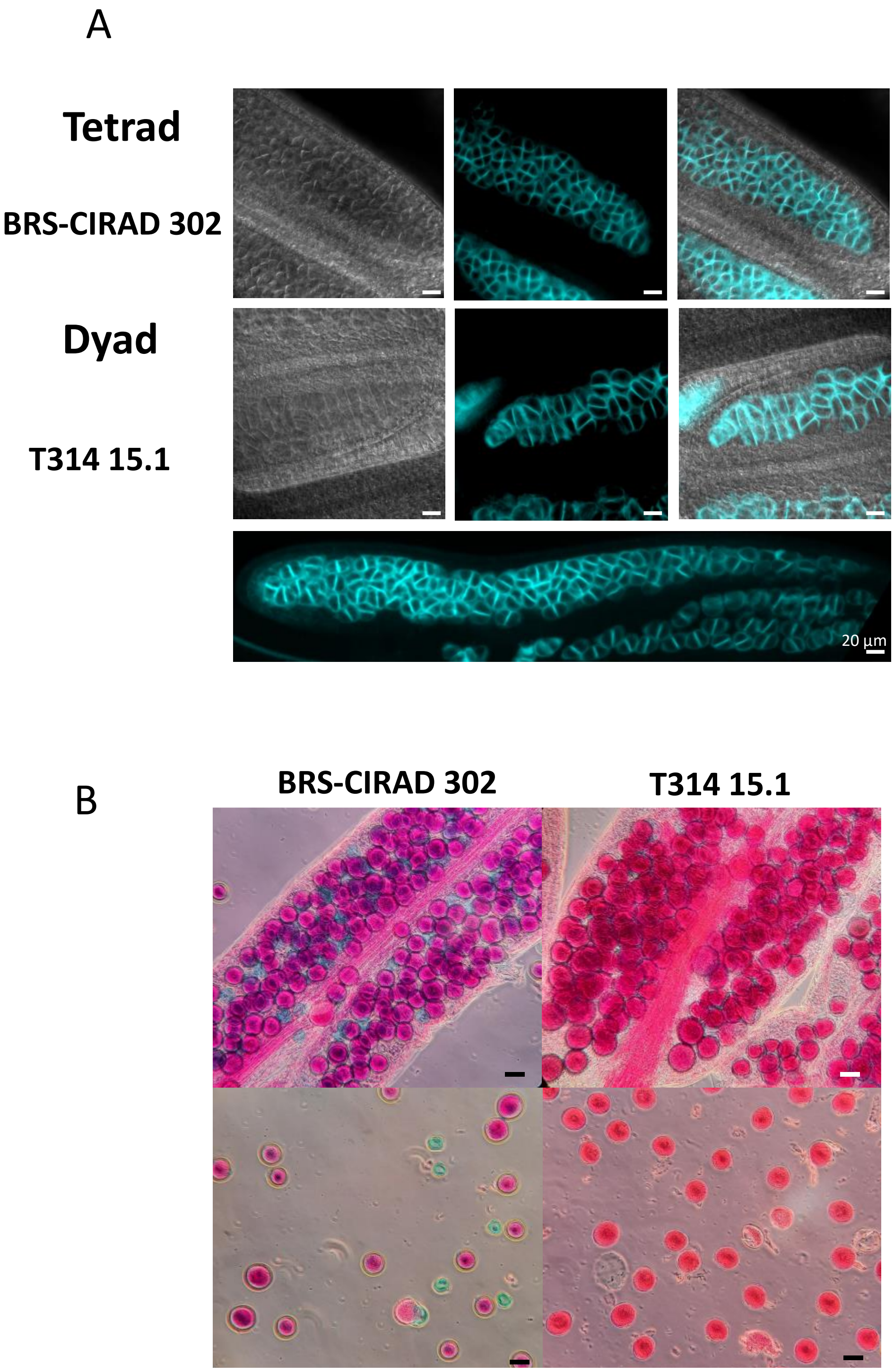

Supplementary Figure 3. Outcome of male meiosis and gametogenesis in apomictic lines. A. Anthers containing tetrads and dyads in BRS CIRAD 302 and T314 15.1 events, respectively. Representative images of callose deposition patterns detected by aniline blue staining (cyan signal, central panel). Similar results were obtained in at least 2 independent plants. Scale bars: 20 µm **B.** Pollen stained with Alexander's solution in representative mature anthers left: BRS-CIRAD 302; right: representative apomictic line (bar=40 µm).

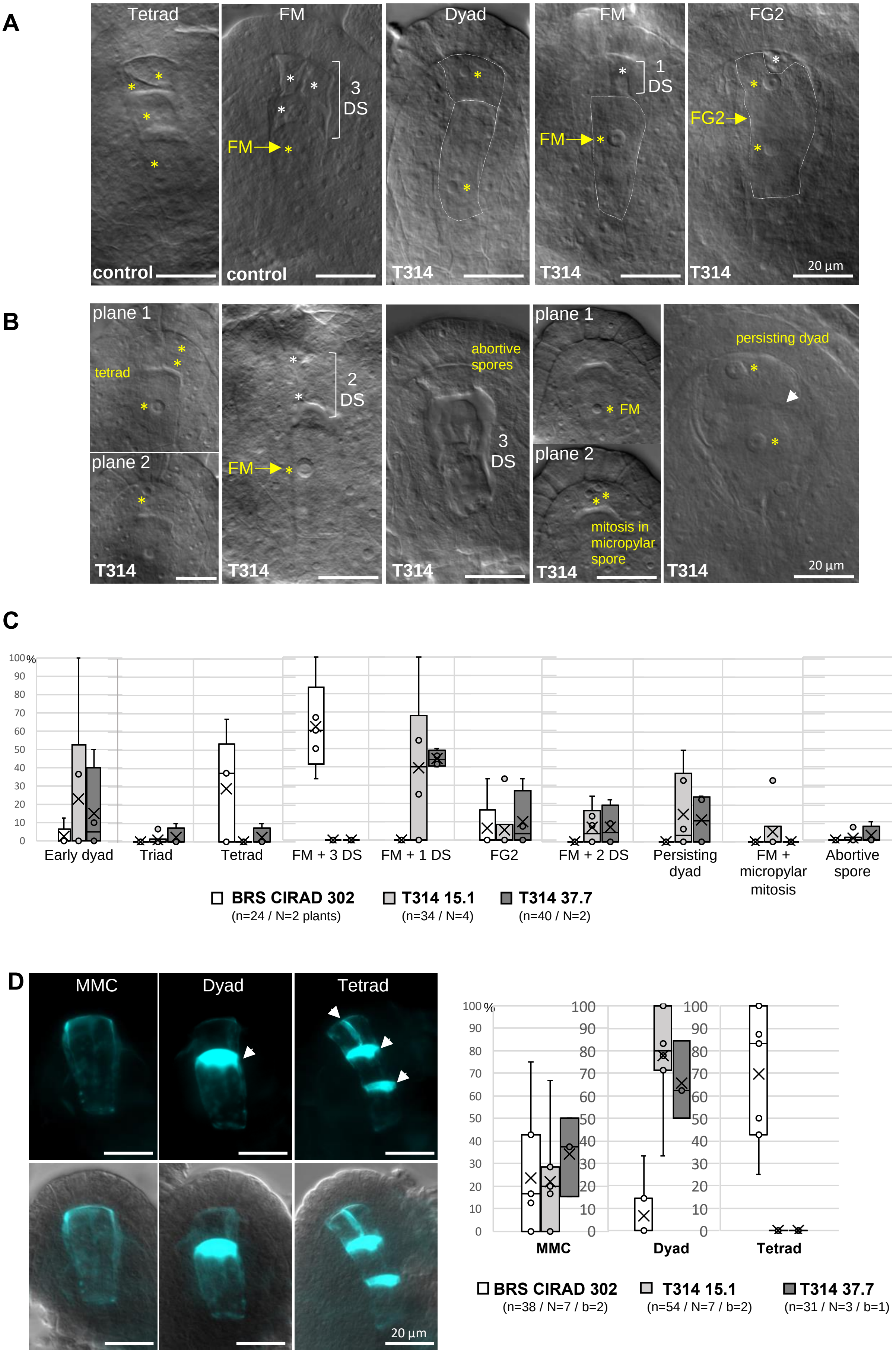
A
Tetrad
FM
Dyad
FM
FG2
3 DS
FM
1 DS
FM
FG2
control
control
T314
T314
T314
20 µm
B
plane 1
tetrad
2 DS
abortive spores
plane 1
persisting dyad
FM
plane 2
FM
3 DS
plane 2
mitosis in micropylar spore
T314
T314
T314
T314
T314
20 µm
C
100 %
Early dyad
Triad
Tetrad
FM + 3 DS
FM + 1 DS
FG2
FM + 2 DS
Persisting dyad
FM + micropylar mitosis
Abortive spore
BRS CIRAD 302 (n=24 / N=2 plants)
T314 15.1 (n=34 / N=4)
T314 37.7 (n=40 / N=2)
D
MMC
Dyad
Tetrad
20 µm
100 %
MMC
Dyad
Tetrad
BRS CIRAD 302 (n=38 / N=7 / b=2)
T314 15.1 (n=54 / N=7 / b=2)
T314 37.7 (n=31 / N=3 / b=1)

Supplementary Figure 4. Cytological analysis of meiotic and post-meiotic ovules in apomictic lines.

A. Representative images of clearing preparations of the major phenotypes observed in control BRS-CIRAD 302 and T314 ovules. Degenerated cells are marked by white asterisks; while functional cells, as assessed by prominent nucleoli and turgescence, are marked by yellow asterisks and dashed lined contours. Scale bars: 20 µm. **B.** Minor phenotypes observed in T314 plants. Symbols as in A. Scale bars: 20 µm. **C.** Quantification of major and minor phenotypes frequencies in BRS-CIRAD 302, T314 15.1, and T314 37.7 samples. Box plots limits represent 2nd and 3rd quartiles, whiskers indicate 1st and 4th quartiles, black line within the box marks the median, cross within the box marks the mean, circles indicate internal and outliers data points. Statistics were derived for (n) ovules, from (N) independent plants - numbers are indicated in chart legend. **D.** Representative images of callose deposition patterns (arrows) detected by aniline blue staining (cyan signal, upper panel) in ovules at MMC, dyad, and tetrad stages. Overlay with cleared tissue is shown in the bottom panel. Scale bars: 20 µm. Box plots (with same elements as described in C.) quantify frequencies of ovules displaying MMC, dyad, or tetrad callose patterns in control plants BRS-CIRAD 302, or T314 15.1, and T314 37.7 plants. Statistics were derived for (n) ovules, from (N) independent plants, from (b) independent batches - numbers are indicated in chart legend.

Abbreviations: FM: functional megaspore; FG2: female gametophyte at 2 nuclei stage; DS: degenerated spore(s); MMC: megaspore mother cell. Scale bars: 20 µm.

Source data is provided as a Source Data file.

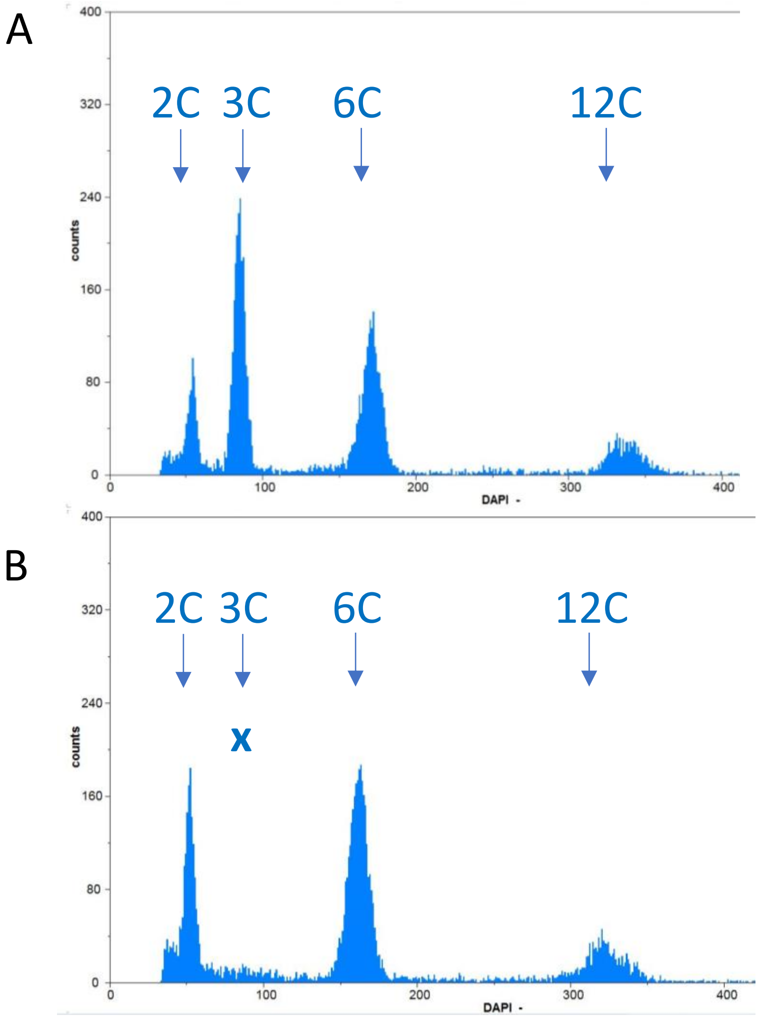

Supplementary Figure 5. Flow cytometry histograms of DAPi-stained nuclei of cells of developing sexual and apomictic endosperms. **A.** control BRS-CIRAD 302 plants; **B.** apomictic plants. Contaminating, nucellar/tegument/pericarp diploid nuclei released with the milky starch endosperm form a 2C peak, providing an internal control in the preparation.

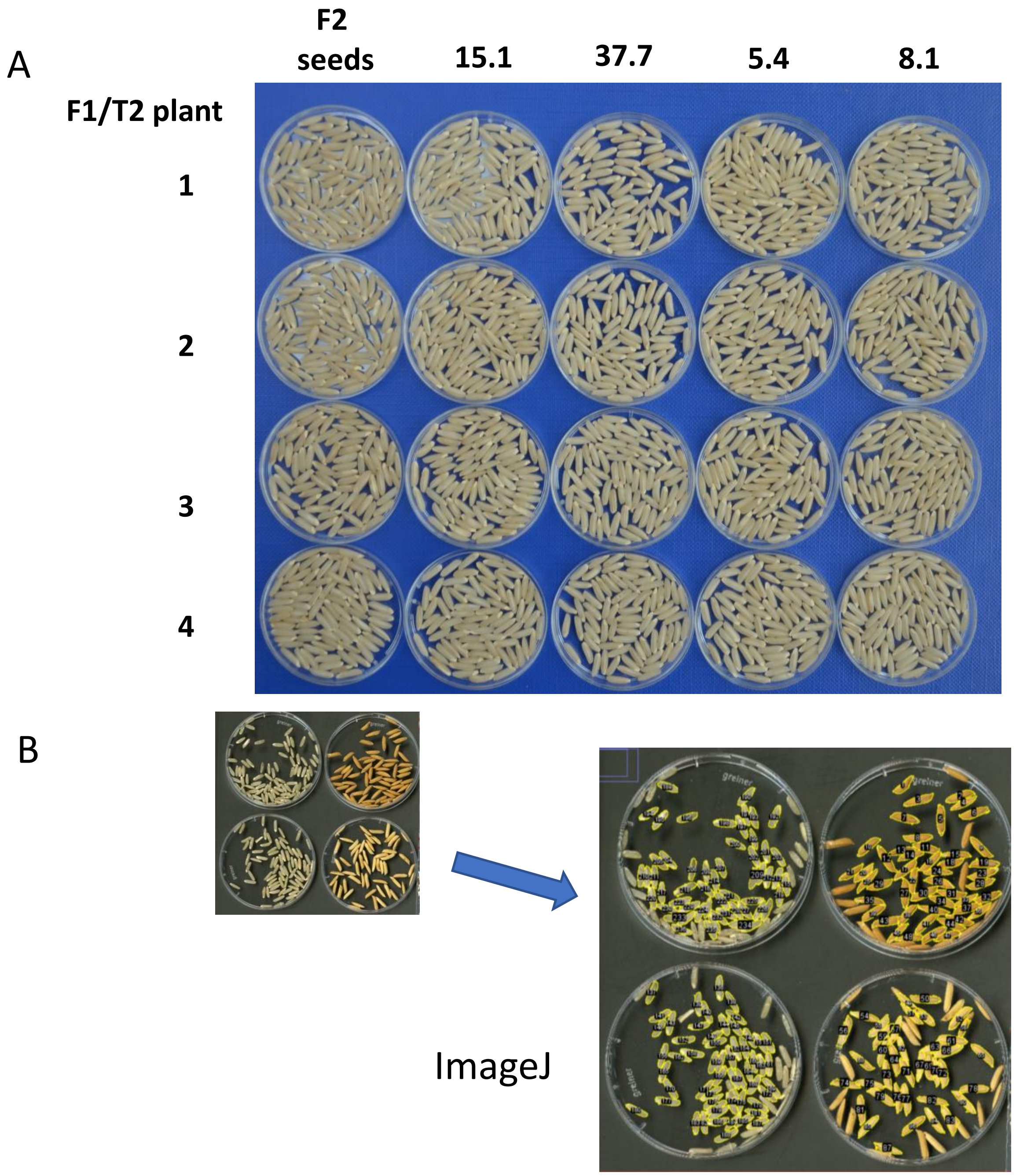

Supplementary Figure 6. Shape of the dehulled mature seeds collected on F1 hybrid and T2 apomictic lines.

A. Dehulled T3 seeds harvested from four independent T2 plants of apomictic events 15.1, 37.7, 5.4, and 8.1 and of F1 BRS-CIRAD 302 used for analysis of seed format. **B.** Snapshot of ImageJ image treatment illustrating the thresholding and the definition of the area of interest for individually numbered kernels, for parental lines 1F (top) and D24 (bottom)

Supplementary reference

[1] Mieulet, D. *et al*. Turning rice meiosis into mitosis. *Cell Research* **26**, 1242-1254 (2016)
