# Supplementary material for: High-frequency synthetic apomixis in hybrid rice
Source: Nat Commun. 2022 Dec 27;13:7963. doi: 10.1038/s41467-022-35679-3 (PMC9794695; doi:10.1038/s41467-022-35679-3)
Supplement: Supplementary file 3 — Reporting Summary [file 41467_2022_35679_MOESM3_ESM.pdf]

nature research

Corresponding author(s): Imltiyaz Khanday
Raphael Mercier
Venkatesan Sundaresan
Emmanuel Guiderdoni

Last updated by author(s): Dec 6, 2022

Reporting Summary

Nature Research wishes to improve the reproducibility of the work that we publish. This form provides structure for consistency and transparency in reporting. For further information on Nature Research policies, see our Editorial Policies and the Editorial Policy Checklist.

Statistics

For all statistical analyses, confirm that the following items are present in the figure legend, table legend, main text, or Methods section.

n/a	Confirmed	
☐	☒	The exact sample size (*n*) for each experimental group/condition, given as a discrete number and unit of measurement
☐	☒	A statement on whether measurements were taken from distinct samples or whether the same sample was measured repeatedly
☐	☒	The statistical test(s) used AND whether they are one- or two-sided<br>*Only common tests should be described solely by name; describe more complex techniques in the Methods section.*
☒	☐	A description of all covariates tested
☐	☒	A description of any assumptions or corrections, such as tests of normality and adjustment for multiple comparisons
☐	☒	A full description of the statistical parameters including central tendency (e.g. means) or other basic estimates (e.g. regression coefficient) AND variation (e.g. standard deviation) or associated estimates of uncertainty (e.g. confidence intervals)
☐	☒	For null hypothesis testing, the test statistic (e.g. *F*, *t*, *r*) with confidence intervals, effect sizes, degrees of freedom and *P* value noted<br>*Give P values as exact values whenever suitable.*
☒	☐	For Bayesian analysis, information on the choice of priors and Markov chain Monte Carlo settings
☒	☐	For hierarchical and complex designs, identification of the appropriate level for tests and full reporting of outcomes
☒	☐	Estimates of effect sizes (e.g. Cohen's *d*, Pearson's *r*), indicating how they were calculated

Our web collection on statistics for biologists contains articles on many of the points above.

Software and code

Policy information about availability of computer code

Data collection	Softwares Flomax v2.9 and Image J v1.53t have been used for flow cytometry and image analyses respectively
Data analysis	For whole genome sequencing analysis, the paired-end raw reads were first quality-evaluated using FastQC v0.11.9 and then mapped to the reference genome using BWA v0.7.15-r1140 with default parameters. Tandem Repeats Finder v4.0953 was used with default parameters to scan the genome tandem repetition of DNA sequences. For the generation of high-confident SNP markers between parental lines, inGAP-family was used with default parameters. The genotype profile was visualized by using ggplot2 v3.3.5 package in R environment. The XLSTAT 2021 v4.1.1204 software (Addinsoft, Paris, France) https://www.xlstat.com/en/ was used for the statistical analyses.

For manuscripts utilizing custom algorithms or software that are central to the research but not yet described in published literature, software must be made available to editors and reviewers. We strongly encourage code deposition in a community repository (e.g. GitHub). See the Nature Research guidelines for submitting code & software for further information.

Data

Policy information about availability of data

All manuscripts must include a data availability statement. This statement should provide the following information, where applicable:

- Accession codes, unique identifiers, or web links for publicly available datasets
- A list of figures that have associated raw data
- A description of any restrictions on data availability

The whole-genome resequencing data of all individuals are available for download in the ArrayExpress database at EMBL-EBI under accession number E-MTAB-11931. https://www.ebi.ac.uk/biostudies/arrayexpress/studies/E-MTAB-11931. The other data that support the findings of this study are provided as Source Data Files. Oryza sativa L. cv Nipponbare genome used for reference was downloaded from Phytozome : https://phytozome-next.jgi.doe.gov/info/Osativa_v7_0

Field-specific reporting

Please select the one below that is the best fit for your research. If you are not sure, read the appropriate sections before making your selection.

☒ Life sciences ☐ Behavioural & social sciences ☐ Ecological, evolutionary & environmental sciences

For a reference copy of the document with all sections, see nature.com/documents/nr-reporting-summary-flat.pdf

Life sciences study design

All studies must disclose on these points even when the disclosure is negative.

Sample size	No statistical test was used to pre determine sample size. The numbers used (@20-30 independent samples) are commonly considered sufficient in experimental plant biology to allow group discrimination in statistical analyses.
Data exclusions	No data was excluded
Replication	We used biological replicates :<br>For T1 generation the number of progenies observed in the containment greenhouse was constrained by the number of seeds available : 15-30 T1 progenies plants were observed per T0 event. For T2 generation, we used at least 6 individual progenies of 5 individual T1 plants for each of the 4 selected T0 events (reaching a minimum n of 34 T2 plants per event).<br>For flow cytometry analysis, the numbers at T1 generation were constrained by number of seeds available. For T2 generation, we used at least 40 progeny plants from 5 randomly chosen individual T1 plants per T0 event (i.e. at least 200 T2 plants). For T3 generation, we used 100 progeny plants from 3 randomly chosen individual T2 plants (i.e. 300 plants per T0 event) which was satisfactory for confidently establishing both germination frequency and frequency of apomixis. When t 2 technical replicates in 5 (endosperm) and 10 (leaves) independent samples were performed they succesfully confirmed the result in all cases.<br>Panicle fertility of apomictic lines at the T2 generation was established from 4 individual T2 progeny plants of 5 randomly chosen T1 plants (i.e. 20 plants per T0 event). For grain shape analyses, we used the T3 seeds harvested on the the latter T2 plants. For amylose content analysis of T3 seeds , 4 biological replicates were prepared per T0 event corresponding to 4 pools of seeds harvested on T2 plants representing 4 individual T1 plants. Biological replicates were also used in pollen viablity (4-6 individual plants) and cytology observations as indicated in the text .
Randomization	Samples were allocated to experimental groups according to their genotype (line and T1 progeny plant). All the materials were chosen at random , notably for photographs. For the greenhouse phenotypic (plant, panicle, seed morphology and fertility) evaluation of T2 progenies and due to constrained space in the containment greenhouse facility, we chose to randomize the individual position of the plants rather than using a randomized block design, which would have permitted to estimate of the environmental covariates. Though not eliminating all the causes of external variation due to the always existing heterogeneity in the artificial environmental conditions of the greenhouse we believe that the variation was at least buffered by such a randomization. Supplementary table 7 shows that there is no significant difference and reduced SD for morphological traits between the 5 T1 progenies derived from the same mother plant indicating that variation was controlled.
Blinding	Blind analysis of samples for seed starch/amylose contents was performed. Blinding to group allocation in other experiments was not relevant .

Reporting for specific materials, systems and methods

We require information from authors about some types of materials, experimental systems and methods used in many studies. Here, indicate whether each material, system or method listed is relevant to your study. If you are not sure if a list item applies to your research, read the appropriate section before selecting a response.

Materials & experimental systems

n/a	Involved in the study	
☒	☐	Antibodies
☒	☐	Eukaryotic cell lines
☒	☐	Palaeontology and archaeology
☒	☐	Animals and other organisms
☒	☐	Human research participants
☒	☐	Clinical data
☒	☐	Dual use research of concern

Methods

n/a	Involved in the study	
☒	☐	ChIP-seq
☐	☒	Flow cytometry
☒	☐	MRI-based neuroimaging

Flow Cytometry

Plots

Confirm that:

☒ The axis labels state the marker and fluorochrome used (e.g. CD4-FITC).

☒ The axis scales are clearly visible. Include numbers along axes only for bottom left plot of group (a 'group' is an analysis of identical markers).

☐ All plots are contour plots with outliers or pseudocolor plots.

☐ A numerical value for number of cells or percentage (with statistics) is provided.

Methodology

Sample preparation	Cell nuclei were isolated from developing leaf blades and seed endosperms by releasing them from tissues into the Sysmex CyStain® UV Ploidy 05-5001 buffer containing DAPI (www.sysmex.de) . For seedlings (control , T1 , T2 and T3) cell nuclei were released by fine manual chopping of leaf blade segments with a razor blade. For endosperm nuclei, the pear-shaped developing seed was gently separated from the lemma and palea and allowed to release its milky endosperm into 0.5 ml of buffer solution using a pipette tip. The cell nuclei suspensions were filtered through a 20 micrometer mesh. For endosperm cell nuclei,, the turbid suspension containing nuclei was diluted in 3 ml of buffer and vortexed before filtering and FACS analysis.
Instrument	PARTEC PAS II Flow cytometer (Partec GmbH, Munster, Germany) Fluorescent excitation lamp at 350nm
Software	FLOMAX Software v2.9 (2014) (Quantum analysis GmbH)
Cell population abundance	There was no cell sorting and purification from filtered preparations of leaf cell nuclei. Importance here is to have a balanced number of nuclei in the different windows and the relative weigth of each windows with regards to the total number of nuclei analyzed.
Gating strategy	Leaf cell nuclei of young BRS CIRAD 302 seedlings were used as diploid control (2C peak) which as set at a 50 or 100 arbitrary fluorescence units and to establish the gate P1 separating the nuclei to be analyzed from the debris. The second gate P2 was established at 12C (leaf) or 16C (endosperm). Gating strategy for determining 2C and 4C peaks is provided in the supplementary information.

☒ Tick this box to confirm that a figure exemplifying the gating strategy is provided in the Supplementary Information.
